# Elucidating the role of RBM5 in osteoclastogenesis: a novel potential therapeutic target for osteoporosis

**DOI:** 10.1186/s12891-023-07002-8

**Published:** 2023-11-29

**Authors:** Yuyang Zhang, Xue Chen, Yuan Xiao, Yibo Mei, Tong Yang, Dongchen Li, Xiaohui Wang, Hao Yang, Dageng Huang, Dingjun Hao

**Affiliations:** 1 Health Science Center, Xi’an Jiaotong University, Xi’an, 710061 China; 2 Translational Medicine Center, Hong Hui Hospital, Xi’an Jiaotong University, Xi’an, 710054 China; 3 Central Hospital, China National Petroleum Corporation, Chengdu, 610051 China; 4 Department of Urology, The First Affiliated Hospital of Xi’an Jiaotong University, Xi’an, 710061 China; 5 Department of Spine Surgery, Hong Hui Hospital, Xi’an Jiaotong University, Xi’an, 710054 China

**Keywords:** RBM5, Osteoporosis, BMD, Osteoclasts differentiation, NFATc1, RAW264.7

## Abstract

Osteoporosis is a prevalent bone disease with multigene involved, and the molecular mechanisms of its pathogenesis are not entirely understood. This study aims to identify novel key genes involved in osteoporosis to discover potential pharmacological targets. We analyzed three microarray datasets and identified four differentially expressed genes. The LASSO model indicated that RNA-binding motif protein 5 (RBM5) is associated with osteoporosis and is a potential drug target. We conducted the Spearman correlation analysis and found 52 genes that were significantly related to RBM5. Enrichment analysis showed that these genes were primarily involved in RNA splicing and osteoclast differentiation pathways. By using lentivirus-based shRNA, we successfully knocked down RBM5 expression in RAW264.7 cell line, which showed that RBM5 knockdown significantly impaired their differentiation potential to mature osteoclasts and significantly inhibited bone-resorbing activity. RT-qPCR analyses revealed the expression of osteoclastogenesis marker genes was downregulated along with RBM5 expression. These findings suggest that RBM5 plays a crucial role in the pathogenesis of osteoporosis and provides a new potential pharmacological target.

**Supplementary Information:**

The online version contains supplementary material available at 10.1186/s12891-023-07002-8.

## Introduction

As human longevity expands, the prevalence of osteoporosis (OP), defined as decreased bone mass density (BMD) and poor bone quality, continues to rise [1]. Even with the slightest impact, bones in people with osteoporosis can break. OP is more common in women than in men and becomes more prevalent after menopause [2]. People with OP have a lifetime fracture risk of up to 40%, and vertebral compression fractures are the most common complication of osteoporosis, constituting a significant aspect of the osteoporotic syndrome [3, 4]. Fractures and the consequent loss of mobility and autonomy often represent a significant reduction in quality of life, and due to the need for hospitalization, osteoporotic fractures of the spine and hip increase the risk of other medical complications.

Osteoclasts are multinucleated giant cells of hematopoietic origin with a unique capacity for bone resorption [5]. Circulating monocytes could be osteoclast progenitors and produce a variety of important bioactive factors for bone metabolism [6]. However, as a multigene-involved disease, the underlying molecular regulated network of dysregulated osteoclasts remains poorly investigated.

In this study, we identified RBM5 highly expressed in peripheral blood mononuclear cells (PBMCs) from patients with OP and involved in the differentiation and function maintenance of osteoclasts, which represents the first study to imply the molecular characteristics and value of RBM5 in OP. Overall, the combination of the initial analysis using public datasets and the subsequent validation experiments in a cell line provided a comprehensive and rigorous approach to investigate the role of differentially expressed genes in osteoporosis pathogenesis.

## Materials and methods

### Public data acquisition and processing

The microarray datasets (GSE2208, GSE7158, GSE56815) were obtained from the NCBI GEO database (http://www.ncbi.nlm.nih.gov/geo/). The expression data were derived from peripheral blood mononuclear cells of both osteoporotic and healthy individuals. We used PBMC samples from patients who had low bone mineral density or peak bone mass in all samples in this study. Those with normal mineral density or peak bone mass were considered healthy. All participants or their families provided informed consent for the use of their data in each GEO dataset, and each study was approved by the Ethics Committee. A total of 68 normal and 67 OP peripheral blood mononuclear cells were obtained from these microarray datasets.

### Common DEGs of PBMCs identification

To analyze the differential gene expression between low and high BMD datasets, we employed the "GEOquery" [7] and "limma" R software packages [8]. *P*-values were adjusted using the Benjamini and Hochberg method. Microarray matrix data underwent normalization with quartiles, while probe summarization was computed using Robust Multi-array Average (RMA). Annotated DEGs were determined via intersection across the three GEO sets, with results visualized using Venn diagrams and upset plots. The "limma" R package facilitated the calculation of differentially expressed genes, with a *P*-value less than 0.05 considered significant. To eliminate batch effects and normalize datasets from different peripheral blood mononuclear cell studies, we utilized the "limma" and "sva" R packages [9]. All analyses were performed in R (v4.1.3) using Bioconductor packages. The GSEA analysis was performed with ClusterProfiler R package [10].

### Construction of LASSO model

Four key differentially expressed genes (DEGs) were identified from all microarray datasets, we then constructed the Least Absolute Shrinkage and Selection Operator (LASSO) model [6]. We reduced the dimensions of the data using the LASSO algorithm analysis with the "glmnet" R software package [11]. The LASSO Cox regression provided the "Coef" of genes as their regression coefficients. We randomly divided the integrated three microarray expression matrices into a training and test set at a ratio of 7:3. The area under the curve (AUC) was calculated using the "pROC" R software package [12].

### Functional enrichment analysis 

We analyzed GO and KEGG pathway enrichment using the online platform gProfiler (version e106_eg53_p16_65fcd97) [13, 14] with BH FDR applying a significance threshold of 0.05. The results were visualized using the "GOplot" package in R software [15]. Enrichment results were displayed only if the minimum number of terms for a gene was greater than 1. Using the "corrgram" R software packages, we visualized the results of Spearman correlation analyses. In addition, we considered Pathways of Gene Set Overrepresentation as significant if FDR was smaller than 0.05.

### Construction of protein–protein interaction 

The STRING database version 11.5 [16] was used to predict the protein interaction network of RBM5. To simplify the complicated PPI network, we used the MCODE plugin in Cytoscape software, and the "CytoHubba" plugin was used to visualize the hub genes in the PPI network.

### RBM5 knockdown in established cell line RAW264.7

RAW264.7 cell line were purchased from Procell (Wuhan, China) and seeded to 24-well plate and maintained in MEM Alpha modification (HyClone, Utah, USA) with 10% FBS (Gibco, Grand Island, NY), cultured at 37 °C and 5% CO2 in an incubator (Thermo Fisher, US). The shRNA sequences for RBM5 (TRCN0000287687) and a negative control shRNA (SHC002) were obtained from the MISSION shRNA Library (Sigma) and subcloned into zsgreen-puro lentivector (Hanbio, Shanghai, China). Transfection was done in compliance with the manufacturer's guidelines. In brief, cells were re-plated at a density of 1 × 10^5^ cells/ml in 24-well plates and allowed to adhere overnight. Subsequently, cells underwent incubation in FBS-free DMEM with shRNA directly (MOI = 30) for 24 h, following which the medium was replaced with complete DMEM and incubated for an additional 24 h. After 72 h, cells were treated with 8ug/ml puromycin (Solarbio, Beijing, China) for 24h to exclude non-infected cells, the selected cells were used for downstream experiments.

### Western blot assays 

Employing RIPA lysis buffer, protein extraction was conducted, subsequently facilitating the separation of a pre-determined quantity of protein through SDS-PAGE. This protein was then transferred onto nitrocellulose membranes. The membranes underwent a sequential incubation procedure with the Rabbit anti-RBM5 Polyclonal Antibody (Absin, Shanghai, China) and corresponding horseradish peroxidase-conjugated secondary antibodies, adhering to stipulated concentrations and durations. The relative gray levels were detected using enhanced chemiluminescence (ECL, NCM, China), and these levels were quantitatively assessed utilizing ImageJ software (Bethesda, USA; version 1.53t).

### RAW 264.7 cells differentiate to osteoclasts

To instigate osteoclast differentiation, both untransfected and transfected RAW 264.7 cells (2 × 10^4^/well in 24-well plate) were suspended in MEM Alpha modification medium (HyClone, Utah, USA), supplemented with RANKL (50 ng/mL, Peprotech, USA). The cultivation process involved seeding the cells onto coverslips, after which they were grown for five days in the absence of RANKL (50 ng/mL). On day 4, cells underwent harvesting for subsequent RT-qPCR analysis, and TRAP staining was performed on day 6. The TRAP staining procedure, conducted post 4% paraformaldehyde fixation, employed a TRAP Stain Kit (Solarbio, Beijing, China) and followed the stipulations in the manufacturer's instructions. Osteoclasts confirmed as TRAP-positive (characterized by the presence of ≥ 3 nuclei) were visualized and photographed utilizing a Leica DM IL inverted phase-contrast microscope (Leica Microsystems, Germany; original magnification 100x). The images were quantified using ImageJ software (Bethesda, USA; version 1.53t).

### Bone resorption assays 

We conducted bone resorption assay to investigate osteoclastic activity. RAW264.7 cells were seeded onto bovine bone slices (JoyTech, Zhejiang, China) and stimulated with RANKL (50 ng/ml) for a ten-day period. Afterward, sonication was used to dislodge cells from the bone slices. These bone slices were subsequently subjected to a gradient ethanol dehydration. For subsequent imaging, the bone slices were coated with gold in a vacuum chamber. We then imaged resorption pits with ZEISS GeminiSEM 360 scanning electron microscope (Jena, Germany; original magnification 27 × and 1,000x) and quantitatively assessed them using ImageJ software (Bethesda, USA; version 1.53t). The analysis involved normalization of the pit area against the entire field area on each individual bone slice.

### Quantitative real-time PCR 

On day 4 of RANKL induction, from six-well plates exhibiting confluency greater than 85%, total RNA was extracted utilizing the RNAsimple total RNA kit (Tiangen, Beijing, China). Subsequently, a standardized quantity of this RNA was employed for reverse transcription, facilitated by the RevertAid First Strand cDNA Synthesis Kit (ThermoFisher, Waltham, USA). Real-time PCR amplification was executed with the SYBR Green qPCR Master Mix (TargetMoi, Boston, USA) using a CFX96™ thermal cycler (Bio-Rad Laboratories). Gapdh RNA levels served as the normalization standard for expression values; the primer sequences employed are detailed in Supplemental Table  S1. Any significant gene expression discrepancies between clones were identified by implementing an unpaired t-test via GraphPad 9.0 (San Diego, USA).

### Statistical analysis

Data analysis and graphical representation were executed using R software (version 4.1.3). Comparative analysis of the two RNA-seq groups was conducted employing the Wilcoxon test, whereas the Pearson analysis was leveraged to ascertain the correlation coefficient among variables. Student's t-test was implemented to contrast the NC and shRBM5 groups. Results were deemed statistically significant for a *P*-value lower than 0.05.

## Results

### Identification and enrichment of DEGs from peripheral blood mononuclear cells

**Fig. 1:**
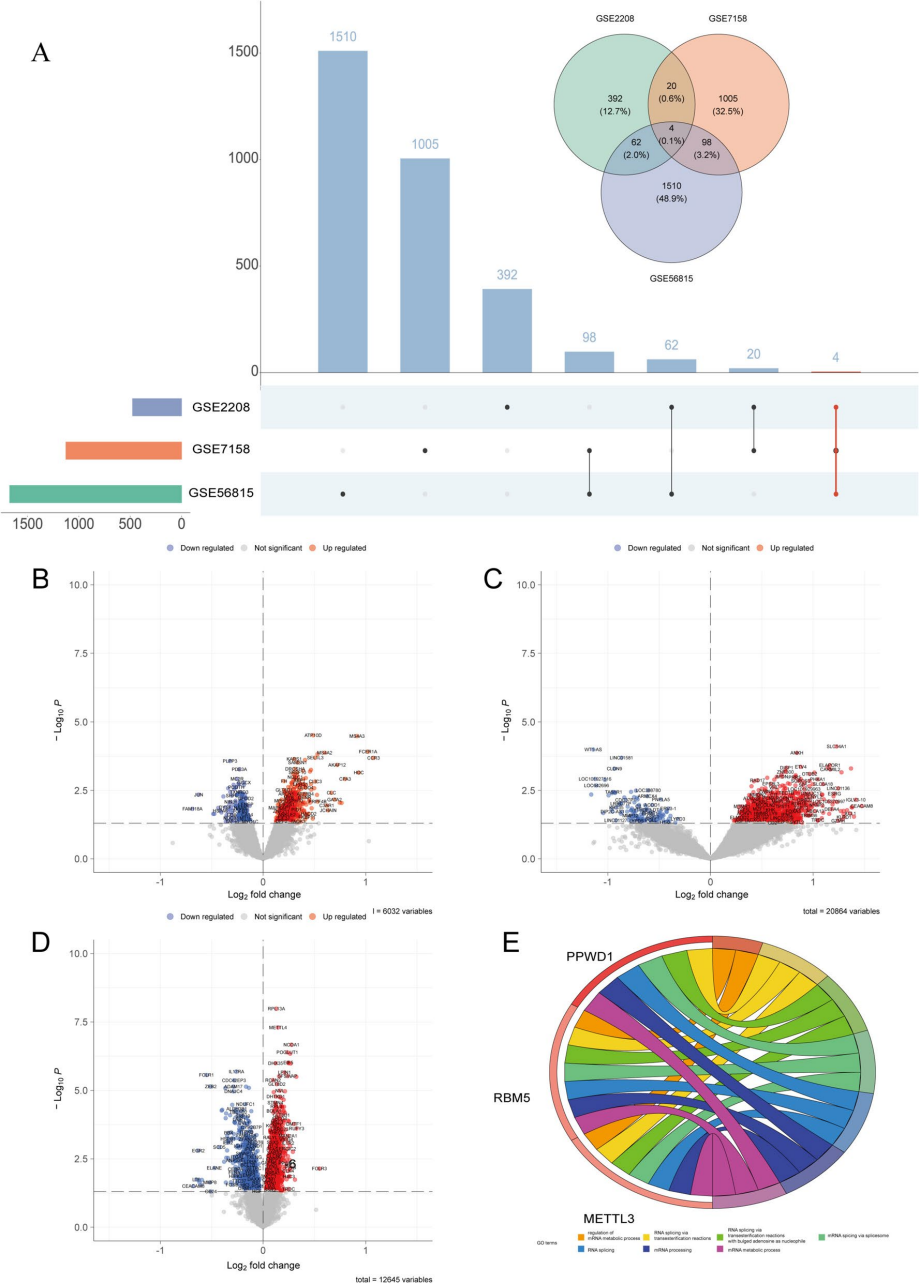
Identification and enrichment of DEGs from peripheral blood mononuclear cells. **A**, Venn diagrams and UpSet plots showed intersections among three GEO datasets. **B**, Volcano plot for GSE2208. **C**, Volcano plot for GSE7158. **D**, Volcano plot for GSE56815. **E**, BP category annotation for intersected DEGs

The aim of this study is to identify differentially expressed genes (DEGs) in peripheral blood mononuclear cell (PBMC) samples to shed light on the possible mechanisms behind osteoporosis (OP) pathogenesis. We utilized three GEO datasets (GSE2208, GSE7158, and GSE56815) as sources of gene expression data (Table 1). Using the “limma” package in R software, we identified DEGs in OP patient cohorts compared to normal individuals. We found 467, 1127, and 1674 up-regulated DEGs and 263, 132, and 803 down-regulated DEGs in the three datasets, respectively. By integrating the results, we identified nine overlapping DEGs in OP patients. After excluding controversially expressed genes, we confirmed four up-regulated DEGs that were conserved in all three datasets (MTMR1, METTL3, PPWD1, and RBM5) (Fig. 1A). Volcano plots were generated to illustrate the profiles of these DEGs in each GEO dataset (Fig. 1B-D).

**Table 1:** Three GEO datasets as sources of gene expression data

**ID**	**GEO**	**Sample Source**	**OP smaples**	**Healthy samples**	**Seq Type**	**Platform**	**Year**	**Country**	**Author**
1	GSE2208	Peripheral blood mononuclear cells	10	9	Micro array	GPL96	2005	USA	Liu YZ, Dvornyk V et al
2	GSE7158	Peripheral blood mononuclear cells	14	12	Micro array	GPL570	2008	China	Lei S, Deng H et al
3	GSE56815	Peripheral blood mononuclear cells	40	40	Micro array	GPL96	2016	USA	Zhou Y, Gao Y et al

To ascertain the roles of the identified DEGs, we applied gene ontology (GO) analysis. We utilized the “GOPlot” package to visualize the enrichment results, which were categorized based on molecular function (MF), biological processes (BP), and cellular components (CC). The BP category contained the largest number of GO terms, and our results indicated that the four DEGs were mainly enriched in RNA splicing pathways, with RBM5 and PPWD1 displaying significant participation in such processes (Fig. 1E). The CC and MF categories demonstrated that these genes were primarily enriched in spliceosomal complex and phosphatidylinositol phosphate activity, respectively (Figures S1A, B).

### Construction of LASSO model for identifying optimal gene marker

**Fig. 2:**
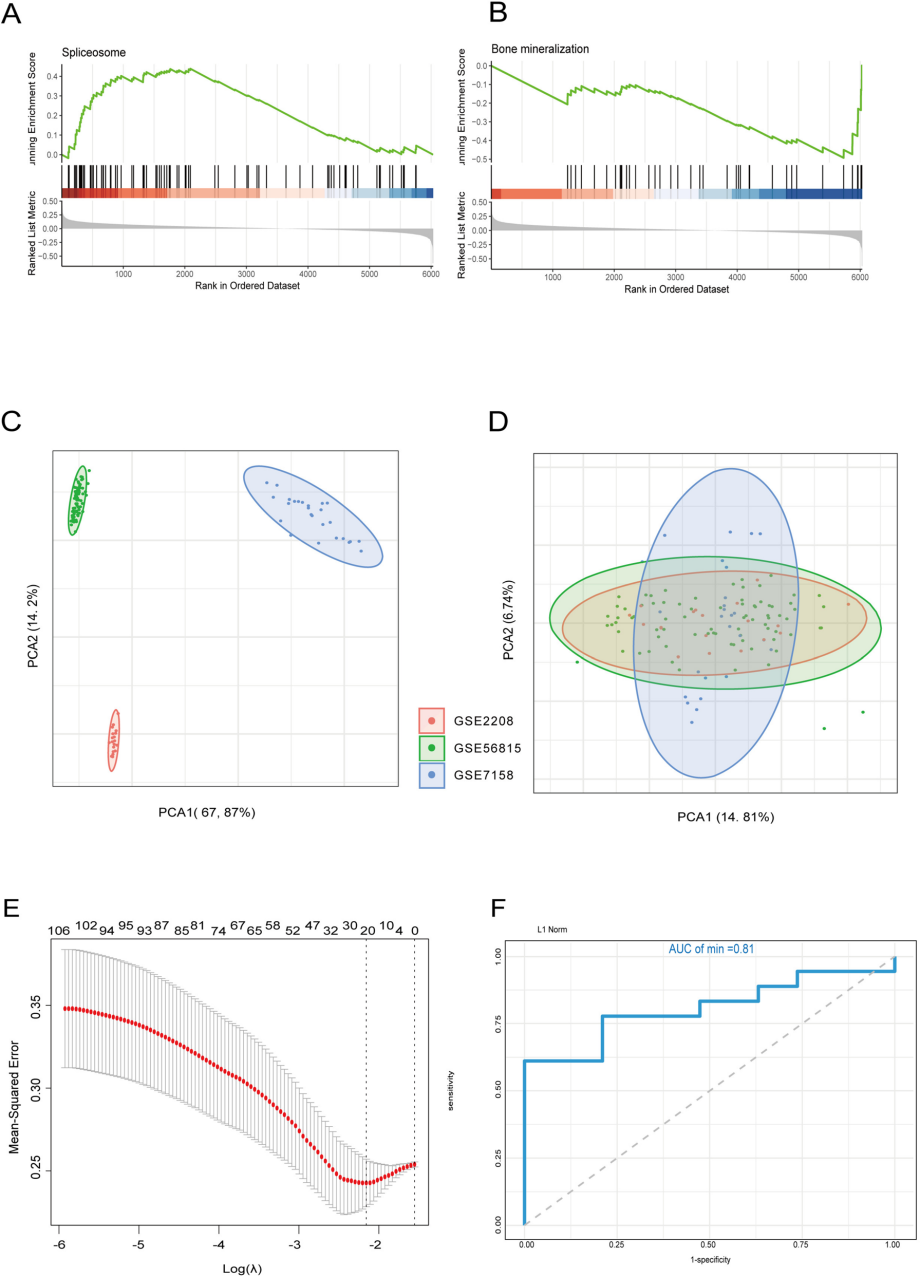
Construction of LASSO model for identifying optimal gene marker. **A**, **B**, GSEA results showed Spliceosome was enriched in High-BMD group while term Bone mineralization was enriched in Low-BMD group. **C**, PCA plot showed batch effect before integration. **D**, Batch effects were eliminated after integration. **E**, Construction of LASSO model. (F), Verification of predictive efficacy of LASSO model, AUC value was 0.81

We performed GSEA (Fig. 2A, B) and PCA analysis (Fig. 2C, D) integrating three microarray datasets. We eliminated batch effects and normalized gene expression, we utilized the LASSO model to select and shrink parameters for identifying genetic markers of OP. LASSO regression analysis on the training and test sets revealed 20 DEGs with non-zero regression coefficients, with a lambda min value of 0.1156 (Fig. 2E, F). The ROC curve showed an AUC of 0.81 in the test set, indicating that the LASSO model could effectively predict the occurrence of OP. Notably, RBM5 was the only gene that intersected with non-zero parameters in the LASSO model, suggesting a strong association with OP and possible involvement in its development. Our findings highlight the potential significance of RBM5 as a genetic marker for OP.

### RBM5 related genes identified by Spearman correlation analysis

**Fig. 3:**
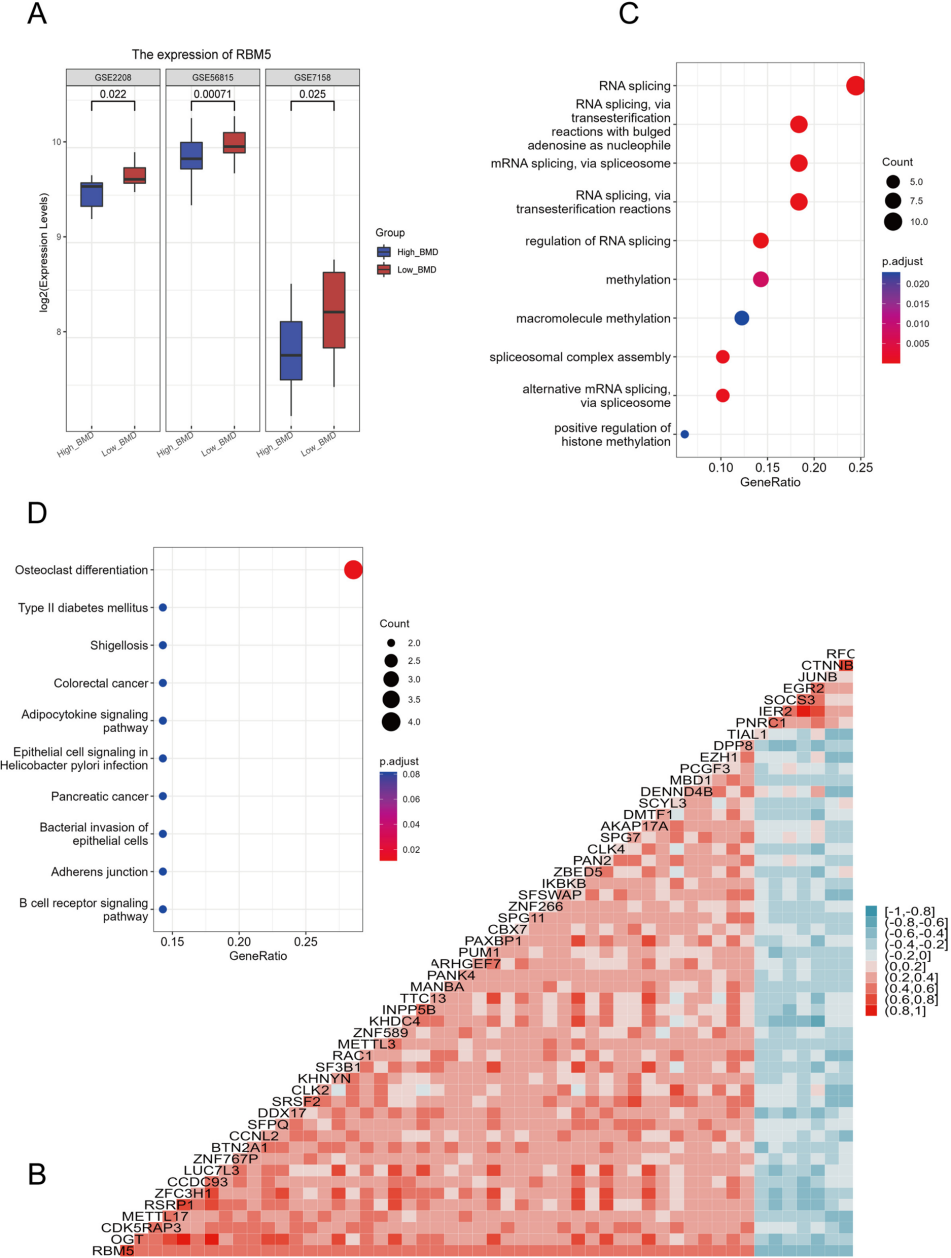
RBM5 related genes identified by Spearman correlation analysis. **A** Box plot revealed that RBM5 was highly expressed in patients with low BMD in all three datasets. **B** 52 genes were identified to be associated with RBM5 expression. **C** GO enrichment for these 53 genes (including RBM5). **D** KEGG enrichment for these 53 genes (including RBM5)

The expression level of RBM5 was found to be consistently higher in all three datasets, as demonstrated by the boxplot (Fig. 3A). However, the precise role of RBM5 in relation to OP remains unclear. Based on our findings, RBM5 appears to be a critical regulatory factor in the development of bone loss. To gain further insight into RBM5's function in OP, we performed enrichment analysis using genes co-expressed with RBM5. Our Spearman analysis identified 52 genes with significant associations with RBM5 (Fig. 3B). Subsequently, we conducted GO and KEGG enrichment analyses to better understand the potential role of RBM5 in the progression of OP. Of the 52 RBM5-associated genes enriched in the GO database, RNA splicing was the most prominent annotation as expected (Fig. 3C). Intriguingly, the only significant term yielded by the KEGG database was related to Osteoclast Differentiation (Fig. 3D).

### Evaluation of protein–protein interaction and hub genes

**Fig. 4:**
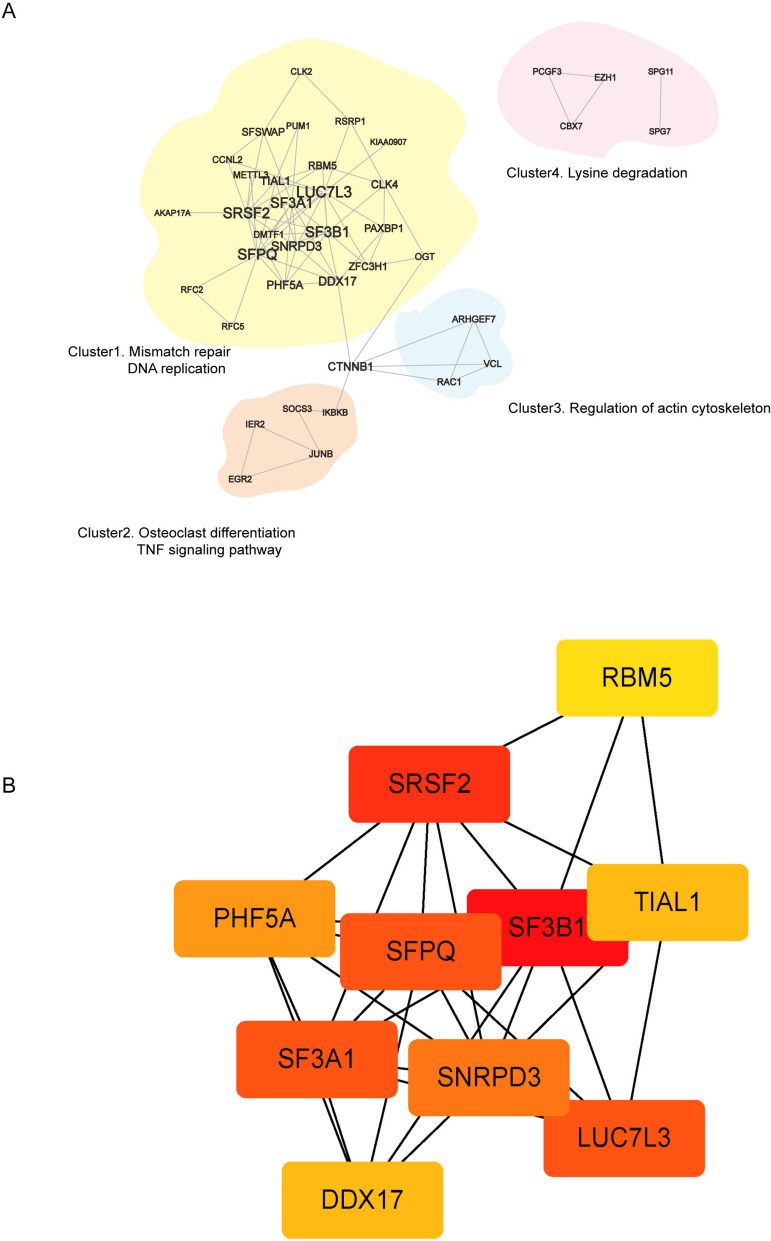
Evaluation of Protein–Protein Interaction and hub genes. **A** PPI network construction and functional annotation. **B** Identification of top 10 hub genes by Cytoscape software

A protein–protein interaction (PPI) network consisting of 53 genes was generated using the Spearman method from the STRING database and visualized in Cytoscape software. The KEGG pathway annotations for these genes were obtained from the gProfiler online platform. The PPI network was divided into four clusters as shown in Fig. 4A: Cluster 1, which was the largest, was highly enriched in Mismatch repair and DNA replication, and considering the biological function of RBM5 itself, the results were not surprising. Cluster 2, which included JUNB, IER2, EGR2, and SOCS3, was annotated to Osteoclast differentiation and TNF signaling pathway. Cluster 3 was associated with the regulation of action cytoskeleton whereas Cluster 4 was related to Lysine degradation.

Furthermore, using the cytoHubba plugin in Cytoscape software, we identified the top 10 ranked hub genes: splicing factor 3b subunit 1 (SF3B1), serine and arginine-rich splicing factor 2 (SRSF2), splicing factor 3a subunit 1 (SF3A1), LUC7 like 3 pre-mRNA splicing factor (LUC7L3), splicing factor proline and glutamine-rich (SFPQ), small nuclear ribonucleoprotein D3 polypeptide (SNRPD3), PHD finger protein 5A (PHF5A), TIA-1-related protein (TIAL1), DEAD-box helicase 17 (DDX17), and RNA binding motif protein 5 (RBM5).

Given that RBM5 was highly expressed in OP patients, we hypothesized that RBM5 might indirectly affect osteoclast differentiation through these correlated genes, especially those in Cluster 2. The top 10 hub genes identified through cytoHubba are shown in Fig. 4B.

### RBM5 knockdown inhibited RANKL-induced osteoclasts differentiation

**Fig. 5:**
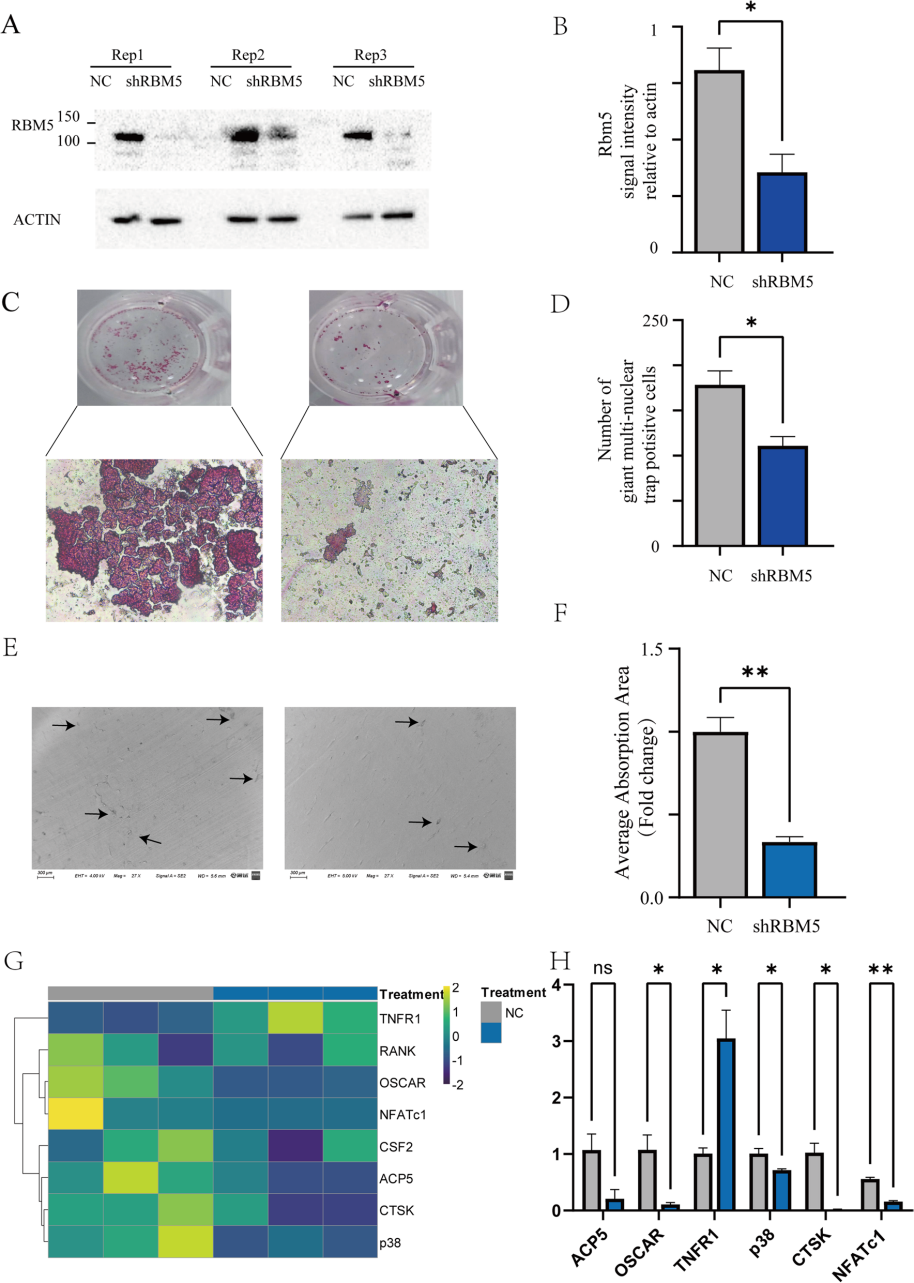
Validation of RBM5 role in osteoclast differentiation. **A**, **B** Lentivirus mediated shRNA knockdown the expression of RBM5 in protein levels. **C**, **D** Representative pictures of TRAP staining for mature osteoclasts induced by RANKL (50ng/ml) for 6 days, positive cells harbored red reaction product in the cytoplasm. **C** 200 × original magnification for lower panel (scale bar: 125 µm). **D** student t test for giant multi-nuclear TRAP positive cells numbers between two groups, *n* = 4. (E–F) resorption pits on the bovine bone slides captured by SEM. Original magnification of 27x (scale bar: 300 µm) for upper panel and 100 × original magnification for lower panel (scale bar: 10 µm). (G-H) Quantification for RT-qPCR results. *P* < 0.05*, *P* < 0.01**, *P* < 0.001 ***determined by the unpaired t-test. ns, no significance

To probe the influence of RBM5 on osteoclast differentiation and function, we conducted RBM5 knockdown in RAW264.7 cells utilizing shRNA (Fig. 5A, B). The substantial decrease in TRAP-positive, typically large, and multinucleated, cells in the RBM5 knockdown group underscores the pivotal role these cells play in bone resorption. The RBM5 knockdown not only led to diminished TRAP-positive cell counts but also resulted in smaller cell size formation (Fig. 5C, D). To validate these findings, we further investigated the bone resorption capacity of osteoclasts on bovine bone slices post-RBM5 knockdown using Scanning Electron Microscopy (SEM). SEM analysis affirmed our preliminary observations and demonstrated that the ability of these RBM5 knockdown cells to resorb mineralized matrix was markedly compromised compared to the control group (Fig. 5E, F). RT-qPCR analyses also unveiled a consequential downregulation of the osteoclast commitment marker genes, including OSCAR (osteoclast-associated receptor), p38, CTSK (Cathepsin K), NFATc1 (Nuclear Factor of Activated T Cells 1) (*P* < 0.05). Interestingly, despite a visible decreasing tendency in the mRNA expression of ACP5 (which encodes TRAP), the changes did not reach statistical significance. In addition, we did not find alterations of RANK expression along with RBM5 knockdown (Fig. 5G, H). These findings collectively suggest that inhibition of RBM5 leads to diminished activation of the p38 pathway. This reduced activation could result in the decreased expression of NFATc1, OSCAR, and CTSK. With NFATc1 and OSCAR being crucial for osteoclast differentiation and CTSK essential for bone matrix degradation, it becomes clear how RBM5 inhibition could impede osteogenesis.

## Discussion

Though much research has been conducted, the regulatory network of differentiation, activation, and apoptosis of osteoclasts in osteoporosis requires further investigation. Identification of biomarker genes in OP PBMCs is, therefore, critical in the understanding of molecular processes underlying the condition and identifying new treatment targets. The aim of this study is to uncover biological markers in OP PBMCs and their potential therapeutic targets, as well as investigate their roles and functions in OP.

Initially, our comprehensive bioinformatics analysis demonstrated a significant association between osteoporosis (OP) and the expression of four specific genes. By implementing the LASSO machine learning approach, we constructed a model and achieved the identification of RBM5. This gene was selected as the only intersecting gene by merging three distinct datasets and detecting non-zero parameters via the LASSO model. The calculated AUC value of 0.81 further reinforced the reliability of the selected RBM5 gene.

RBM5 is a protein composed of 815 amino acids and functions as a component of the spliceosome A complex, being able to regulate mRNA alternative splicing. RBM5 was initially denominated “LUCA15” [17], with its name later being changed to “RNA-binding protein 5” by HGNC. Previous research has suggested that RBM5 may act as a potential tumor suppressor gene [18–20], and that it could positively and negatively regulate apoptosis through alternative splicing of various genes, such as FAS and CASP2/caspase-2 [21], thus proposing its involvement in the progression of apoptosis. Nonetheless, RBM5’s connection to OP remains an underexplored topic. To gain more insights into the roles of the four genes associated with OP, we analyzed their protein–protein interaction network by utilizing the STRING database.

Currently, the relationship between RBM5 and osteoporosis has not been investigated. However, a recent study has shed light on the potential connection between the two. The study found that overexpressed RBM5-AS1 inhibits apoptosis of both osteoblasts and chondrocytes in animal fracture models [22]. Since RBM5-AS1 acts as the natural antisense transcript of RBM5, it is logical to infer that overexpressed RBM5 may promote apoptosis of osteoblasts and chondrocytes. This finding could also explain the observed upregulation of RBM5 expression in PBMC of osteoporosis patients.

Further in vitro experiments were performed using the RAW264.7 cell line, a well-established robust model system for osteoclast differentiation and function [23, 24]. While M-CSF is indispensable for osteoclast differentiation from primary monocytes or bone marrow-derived macrophages, its requirement can be bypassed when using the RAW264.7 cell line. This characteristic allowed us to streamline our differentiation and bone resorption assays and focus more on the RANKL/RANK pathway in osteoclastogenesis. The use of this cell line allowed for more specific and controlled experiments that provided invaluable insights into the molecular underpinnings of osteoclast differentiation and function.

The significance of RBM5 knockdown in the realm of osteoclastogenesis cannot be understated. As our results have elucidated, RBM5 knockdown bears an influence on the p38 MAPK pathway and consequently NFATc1 and its downstream. One key piece of evidence bolstering this assertion is the observed reduction in osteoclastogenesis following RBM5 knockdown, as manifested in the decreased number of TRAP-positive multinuclear cells. Further compounding this observation is the reduced bone resorption abilities of osteoclasts, demonstrated vividly in SEM-scanned bovine bone slices.

The p38 MAPK pathway is a crucial signaling cascade renowned for its role in governing cellular responses to a diverse range of external stressors, from inflammatory cytokines to physical stimuli [25]. Its involvement spans a plethora of cellular processes, notably differentiation, apoptosis, and inflammatory reactions [26, 27]. This pathway, known for orchestrating a plethora of cellular responses to external stressors, plays an indispensable role in bone homeostasis, particularly in the context of osteoclastogenesis. The p38 MAPK pathway acts as a regulatory hub, intricately linked with the modulation of NFATc1 [28], a transcription factor vital for osteoclast differentiation [29]. NFATc1's role in this biological dance is to coordinate the expression of a wide array of osteoclast-specific genes [30]. This coordination ensures that osteoclasts not only mature appropriately but also function with optimal efficacy.

Our results indicate that activation of the p38 MAPK pathway is inhibited when RBM5 is knocked down. The perturbation of the p38 MAPK pathway directly influences the expression of NFATc1, which orchestrates the gene expression necessary for osteoclasts' maturation and bone-resorbing activity. Thus, any modulation in the activity of the p38 MAPK pathway, such as that instigated by RBM5 knockdown, directly reflects in NFATc1 expression levels. As NFATc1 expression wanes, it takes with it the optimal expression of its downstream targets, including the enzyme CTSK (cathepsin K) and OSCAR (osteoclast-associated receptor). CTSK's role in bone resorption is crucial [31]; it facilitates the degradation of the bone matrix, a process vital for the normal functioning of mature osteoclasts [32]. As a crucial enzyme responsible for bone matrix degradation, any disruption in CTSK levels can be linked directly to compromised osteogenesis. OSCAR serves as a potent costimulatory receptor, as demonstrated in vitro, and its gene expression in mice is induced by RANKL, positioning it within the later stages of preosteoclast maturation [33]. The intricate web of molecular interactions governing osteoclastogenesis becomes particularly pronounced upon the knockdown of RBM5. Within this framework, two entities emerge as central figures: the p38 MAPK pathway and the transcription factor NFATc1. A systematic exploration of their interrelationship elucidates the complexities underpinning osteoclast differentiation and function.

In addition, in our study, we observed an unexpected increase in TNFR1 mRNA expression following the knockdown of RBM5. This is particularly intriguing given the reported promoting role of TNFR1 on osteoclastogenesis. One possible explanation for this observed phenomenon could be the initiation of a compensatory response within the cells. It can be hypothesized that in the face of RBM5 suppression, the cells may seek to restore osteoclast differentiation and function by increasing the expression of TNFR1. However, such an interpretation should be approached with caution, as the precise biological functions of RBM5 are complex and not entirely understood. RBM5 is known to regulate apoptosis and alternative splicing of various genes [34, 35] and its downregulation could potentially disrupt the normal function of other signaling pathways, leading to an indirect increase in TNFR1 expression. It is also worth noting that cellular signaling pathways are often characterized by intricate feedback loops and cross-communication, suggesting that the relationship between RBM5 and TNFR1 might be influenced by a multitude of factors and might not be linear [36, 37]. Thus, the functional implications of the observed increase in TNFR1 mRNA expression following RBM5 knockdown warrant further investigation.

In conclusion, the trajectory from RBM5 knockdown unfolds as follows: The downregulation of RBM5 suppresses the p38 MAPK pathway, which in turn impacts NFATc1 expression. This sequential disruption culminates in the modulation of osteoclast markers, with profound implications for bone health. We must acknowledge that the RAW264.7 cell line utilized in this study could only partially reflect osteoclastogenesis process in vitro and further research based on primary cell culture and in vivo experiments could elucidate the intricate relationships between these molecules and pathways, paving the way for therapeutic strategies targeting bone-related disorders. This comprehension could form the basis for innovative therapeutic strategies targeting bone diseases, with RBM5 serving as a potential therapeutic target. Such therapeutic interventions could provide a novel approach to OP management, presenting new avenues for further investigation.

## Conclusions

This study found that RBM5 is overexpressed in osteoporosis patients. RBM5 expression knockdown in the RAW264.7 cell line impairs its ability to differentiate into osteoclasts in vitro, which may be mediated through the p38/NFATc1 signaling pathway.

### Supplementary Information

**Additional file 1:** **Table S1.** The primers used for RT-qPCR analysis.

**Additional file 2: Figure S1.** CC and MF category annotation for intersected DEGs.

## Data Availability

The datasets analyzed for this study are available in the GEO database [https://www.ncbi.nlm.nih.gov/geo] with accession numbers GSE2208, GSE7158, and GSE56815.

## Abbreviations

OP: Osteoporosis
BMD: Bone mineral density
OC: Osteoclasts
TRAP: Tartrate-resistant acid phosphatase
DEGs: Differentially expressed genes
PCA: Principal component analysis
GO: Gene Ontology
AUC: Area under curve
ROC: Receiver operator characteristic curve
SEM: Scanning electron microscope
