# Supplementary figures and images for: Elucidating the role of RBM5 in osteoclastogenesis: a novel potential therapeutic target for osteoporosis
Source: BMC Musculoskelet Disord. 2023 Nov 29;24:921. doi: 10.1186/s12891-023-07002-8 (PMC10688468; doi:10.1186/s12891-023-07002-8)

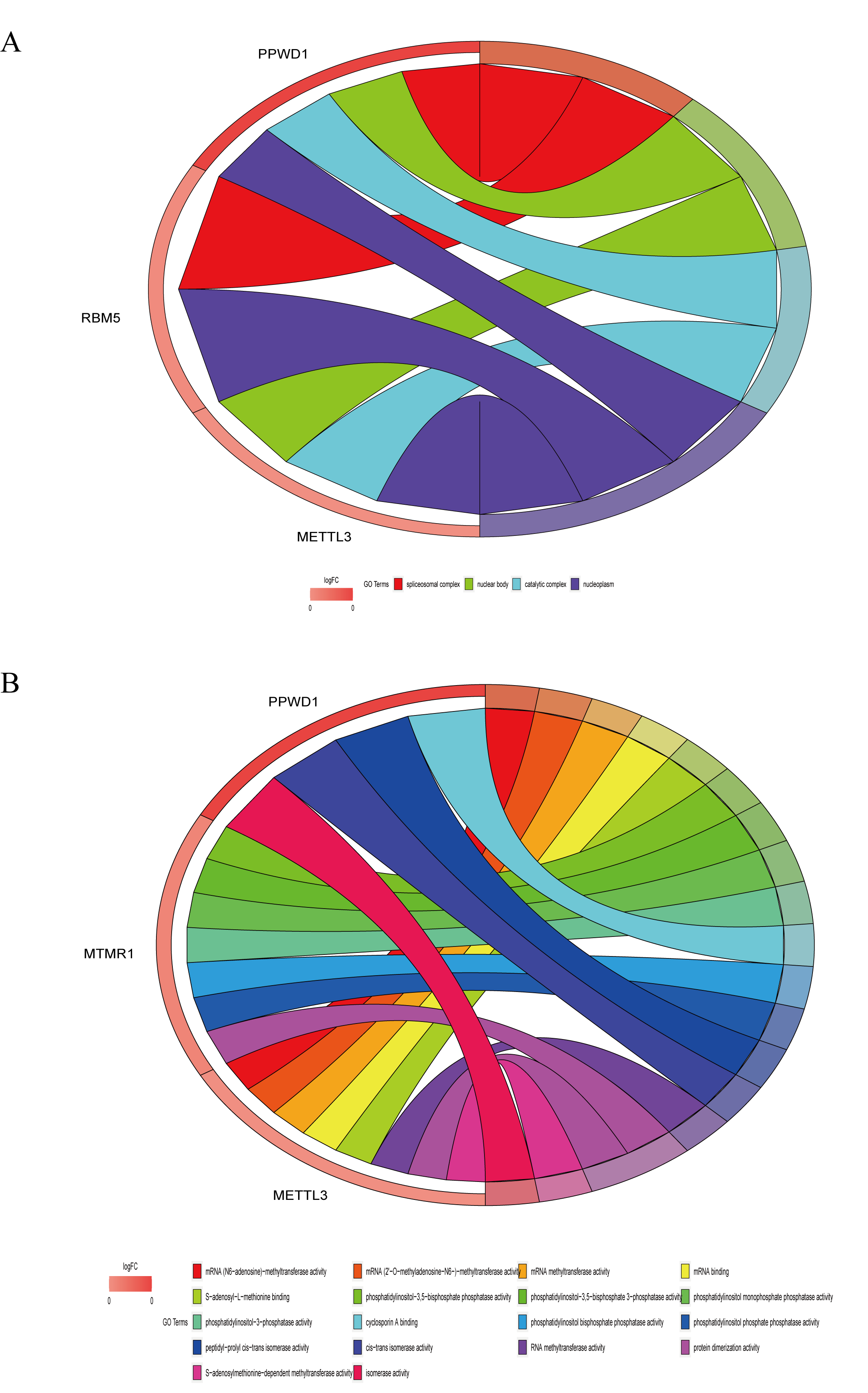

Supplement: Supplementary file 2 — Additional file 2: Figure S1. CC and MF category annotation for intersected DEGs. [file 12891_2023_7002_MOESM2_ESM.tif]
